# A real-world pharmacovigilance study of adverse events associated with esketamine: disproportionality analysis and detection of potential drug-drug interaction signals

**DOI:** 10.1007/s00228-025-03954-z

**Published:** 2025-12-22

**Authors:** Claudia Pisanu, Shungo Imai, Masami Tsuchiya, Mari Inoue, Keisuke Ikegami, Gianpaolo Zammarchi, Hayato Kizaki, Satoko Hori

**Affiliations:** 1 Department of Biomedical Sciences, Section of Neuroscience and Clinical Pharmacology, University of Cagliari, Cagliari, Italy; 2 Division of Drug Informatics, Keio University Faculty of Pharmacy, Tokyo, Japan; 3 Department of Economics and Business Science, University of Cagliari, Cagliari, Italy

**Keywords:** Esketamine, Adverse drug reaction, Adverse event, Drug-drug interaction, FAERS, Depression

## Abstract

**Purpose:**

We conducted a comprehensive analysis of esketamine-related adverse events (AE) on the FDA Adverse Event Reporting System (FAERS) database, taking into account for the first time drug-drug interaction signals.

**Methods:**

We conducted a retrospective case/non-case study of esketamine-related AEs reported in the FAERS database up to the last quarter (Q4) of 2024. Potential signals were detected using the reporting odds ratio (ROR) and confidence intervals (CI), while drug-drug interactions were studied using different metrics such as lift, conviction and the combination risk ratio detection algorithm. An analysis of sex differences was also performed using the relative ROR and CI.

**Results:**

The analysis of 7,790 reports in which esketamine was a primary or secondary suspect identified potential safety signals for 173 AEs. Novel signals include homicidal ideation (ROR = 5.30, 95% CI: 2.38-11.82) and substance use disorder (ROR = 6.12, 95% CI: 2.54-14.73). Women showed a longer time to onset than men (p = 0.003). In addition, we detected sex differences in 23 AEs, seven of which were more likely to be reported in women, while 16 in men. Among these, four were significant exclusively in women (oxygen saturation decreased, abnormal behaviour, unresponsive to stimuli and aggression) and two in men (vision blurred and bradycardia). Potential signals of additive and multiplicative drug-drug interactions were detected for antidepressants (venlafaxine for“dizziness” and bupropion for “agitation”) and antipsychotics (risperidone for “vertigo”).

**Conclusions:**

Our results increase knowledge on potential risks related to esketamine AEs and potential drug-drug interaction signals in a real-world setting.

**Supplementary Information:**

The online version contains supplementary material available at 10.1007/s00228-025-03954-z.

## Introduction

Major depressive disorder (MDD) is a substantial determinant of clinical and socioeconomic burden [1, 2]. While pharmacological treatment with antidepressants is the mainstay in the management of MDD, there is a large interindividual variability in clinical response, with around one third of patients being diagnosed with treatment-resistant depression (TRD). Intranasal esketamine has been approved by the Food and Drug Administration (FDA), the European Medicines Agency and regulatory entities in several countries as an adjunct to oral antidepressant therapy for patients with TRD or patients with MDD with acute suicidal behaviors or ideation [3] due to its rapid antidepressant effects at subanesthetic doses [4]. Esketamine is the (S) enantiomer of ketamine and acts as a non-selective, non-competitive N-methyl-D-aspartate (NMDA) receptor antagonist [5]. A Risk Evaluation and Mitigation Strategy program was mandated to achieve a better risk-benefit balance, in light of the esketamine potential for misuse and abuse and potential adverse events (AE) such as dissociation and sedation. Nonetheless, concerns have been raised with respect to safety and tolerability [3]. Spontaneous reporting systems are a relevant source of data for the identification of AEs in the real-world setting [6]. Studies that have investigated tolerability of esketamine using data from the largest publicly available pharmacovigilance database, the FDA Adverse Event Reporting System (FAERS) confirmed potential safety signals for frequent AEs such as dissociation and sedation, and raised awareness on rarer AEs such as ataxia or akathisia [7]. Guo and colleagues focused on neurological esketamine-related AEs reported from 2019 to 2021 [8], while the most recent study analyzed AEs reported from the first quarter (Q1) of 2019 to the last quarter (Q4) of 2023 [9]. Other recent studies focused on specific AEs such as alcohol and substance misuse [10, 11]. While all studies found that female outnumber male patients in reports related to esketamine AEs [12], one study reported women to be more likely than men to suffer from serious AEs [7] while another found no significant sex differences [8]. A study specifically focused on the analyses of sex differences, that included reports to the second quarter (Q2) of 2023 suggested some AEs such as completed suicide, decreased therapeutic product effects, urinary retention and hypertension to be more frequent in men [13]. Since January 2025, FDA has also approved esketamine as a monotherapy for adults with TRD [14]. However, since esketamine is often used as an adjunct to oral antidepressant therapy, the identification of drug-drug interaction signals is of high interest. In previous studies, patients with serious AEs were more likely to receive antidepressant polypharmacy, benzodiazepines or somatic medications than patients with non-serious AEs [7, 8]. However, to our knowledge, no study investigated which drugs might interact with esketamine to increase the probability of specific AEs. In this study we provide an updated picture of esketamine related AEs based on a comprehensive analysis of the FAERS database. We conducted stratified analyses based on sex and seriousness and, for the first time, we conducted an in-depth analysis to identify concomitant drugs that might increase the risk of specific AEs.

## Materials and methods

### Data source and extraction

We conducted a real-world pharmacovigilance study based on a disproportionality analysis including individual case safety reports of AEs recorded in the FAERS database over the period 2014 Q3–2024 Q4. FAERS data are organized in quarterly data extracts, each of which includes reports received by FAERS during one quarter of the year. AEs were coded based on the preferred term (PT) codes from the Medical Dictionary for Regulatory Activities [15]. Files containing demographic and administrative information, drug information, PTs, outcomes, therapy start/end dates and indications were downloaded. In case duplicate CASEIDs were identified, the most recent FDA_DT (the data in which the FDA received the case) or the highest PRIMARYID (in case the same CASEID and FDA_DT were identified) were kept, according to FDA recommendations [16, 17]. Reports with the same values for sex, age, country, event date, PT, drug and indication were also identified as duplicates [18]. Drug names reported only as brand names were converted into active ingredients based on the DiAna dictionary [19]. Cases listed in the FAERS deleted files were removed as recommended [20]. We used a case/non case study design comparing AEs in which esketamine was coded as primary suspect (PS) or secondary suspect (SS) against AEs involving all other drugs. Cases in which esketamine was not coded as PS or SS, or cases in which indications for esketamine were not related to psychiatric disorders (e.g. anesthesia, sedation or pain) were excluded. Reporting was conducted according to the READUS-PV guidelines [21].

### Statistical analysis

We calculated reporting odds ratios (ROR) [22] and 95% confidence intervals (CI) as in:

$$\:\mathrm{R}\mathrm{O}\mathrm{R}=\:\frac{\left(\frac{\mathrm{a}}{\mathrm{c}}\right)}{\left(\frac{\mathrm{b}}{\mathrm{d}}\right)\:}\:\:\:\:\:\:\:\:\:\:\:95\mathrm{\%}\:\mathrm{C}\mathrm{I}=\:{\mathrm{e}}^{\mathrm{ln}\left(\mathrm{R}\mathrm{O}\mathrm{R}\right)\pm\:1.96\:\sqrt{\frac{1}{\mathrm{a}}+\:\frac{1}{\mathrm{b}}\:+\:\frac{1}{\mathrm{c}}\:+\:\frac{1}{\mathrm{d}}\:}}$$

where *a* is the number of reports of a target AE with esketamine, *b* the number of reports with other AEs with esketamine, *c* the number of target AEs with all the other drugs, and *d* the number of other AEs with all the other drugs. ROR and 95% CIs were computed for AEs with at least five reports to reduce the risk of false positives [8, 23]. A signal was considered when the lower limit of the 95% CI exceeded 1. A sensitivity analysis was carried using the second-line antidepressant agent venlafaxine as comparator as previously done in the first study evaluating esketamine-related safety signals in FAERS [7]. In order to investigate sex differences in potential signals, the relative RORs based on sex-stratified RORs for women and men were also calculated. For AEs for which at least 5 reports for both women and men were available, we computed relative RORs and 95% CIs as in:

$$\:\mathrm{R}\mathrm{e}\mathrm{l}\mathrm{a}\mathrm{t}\mathrm{i}\mathrm{v}\mathrm{e}\:\mathrm{R}\mathrm{O}\mathrm{R}\:\mathrm{W}\mathrm{o}\mathrm{m}\mathrm{e}\mathrm{n}=\:\frac{{ROR}_{women}}{{ROR}_{men}}\:\:\:\:\:\:\:\:\mathrm{R}\mathrm{e}\mathrm{l}\mathrm{a}\mathrm{t}\mathrm{i}\mathrm{v}\mathrm{e}\:\mathrm{R}\mathrm{O}\mathrm{R}\:\mathrm{M}\mathrm{e}\mathrm{n}=\:\frac{{ROR}_{men}}{{ROR}_{women}}\:\:\:\:\:\:\:\:$$

$$\begin{aligned}&\\&95\mathrm{\%}\:\mathrm{C}\mathrm{I}=&\\&{\mathrm{e}}^{\mathrm{ln}\left(\mathrm{r}\mathrm{e}\mathrm{l}\mathrm{a}\mathrm{t}\mathrm{i}\mathrm{v}\mathrm{e}\mathrm{R}\mathrm{O}\mathrm{R}\right)\pm\:1.96\:\sqrt{\left({\frac{1}{a}}_{women}+{\frac{1}{b}}_{women}+{\frac{1}{c}}_{women}+{\frac{1}{d}}_{women}\right)+\left({\frac{1}{a}}_{men}+{\frac{1}{b}}_{men}+{\frac{1}{c}}_{men}+{\frac{1}{d}}_{men}\right)}}\end{aligned}$$

Women were considered more likely to report an AE in case the lower bound of the 95% CI of ROR_women_ was greater than 1 and the lower bound of the 95% CI of the relative ROR women was greater than 1, while men were considered more likely to report an AE in case the lower bound of the 95% CI of ROR_men_ was greater than 1 and the lower bound of the 95% CI of the relative ROR men was greater than 1.

A subgroup analysis was also conducted to investigate potential differences between adults (between 18 and 64 years old) and older adults (≥ 65 years old). The analysis only included participants for which age was reported in years (*n* = 4,704 adults and *n* = 477 older adults). For AEs for which at least 5 reports for both age groups were available, we computed relative RORs and 95% CIs as described for the sex stratified analysis. Older adults were considered more likely to report an AE in case the lower bound of the 95% CI of ROR_older adults_ was greater than 1 and the lower bound of the 95% CI of the relative ROR in older adults was greater than 1.

For cases for which both dates were available (*n* = 2,599), time-to-onset (TTO) was computed as the difference between the date in which the treatment was started and the date in which the event occurred or started. TTO was compared between men and women with the Mann-Whitney U test. In addition, we compared characteristics between serious and non-serious reports. Serious cases were defined in accordance with the definition of the FDA [24] as those listed in the OUTC FAERS table due one or more of the following outcomes: death, hospitalization, life-threatening, disability, congenital anomaly, required intervention to prevent permanent impairment/damage and/or other serious outcomes. Median age, weight and TTO were compared between serious and non-serious cases with the Mann-Whitney U test, while sex with Pearson’s chi-squared test.

After exclusion of PTs overlapping with the underlying diagnosis or indications for esketamine (anxiety, depression, panic attack, completed suicide, suicidal ideation and suicide attempt), drug-drug interaction analyses were conducted on eight AEs with a significant safety signal for esketamine and for which concomitant drugs were reported in at least three cases, as in previous studies [25], as PS, SS or interacting drugs (drugs only reported as “concomitant” were excluded). We used an association rule mining (ARM) model, an unsupervised learning method that allows to detect relationships between variables in a dataset. To evaluate the degree of the influence of drug B on drug A induced AE, an association rule can be established as:

B → A ∩ AE.

where B is drug B and A ∩ AE is the AE induced by drug A [25]. We used the two metrics lift and conviction to evaluate the strength of drug-drug interaction signals. Lift measures the relative magnitude of the probability of observing A ∩ AE under the condition of drug B, compared to the probability of observing A ∩ AE:

Lift (B → A ∩ AE) = $$\:\frac{\mathrm{C}\mathrm{o}\mathrm{n}\mathrm{f}\mathrm{i}\mathrm{d}\mathrm{e}\mathrm{n}\mathrm{c}\mathrm{e}\:\left(\mathrm{B}\:\to\:\:\mathrm{A}\:\cap\:\:\mathrm{A}\mathrm{E}\right)}{\mathrm{S}\mathrm{u}\mathrm{p}\mathrm{p}\mathrm{o}\mathrm{r}\mathrm{t}\:(\mathrm{A}\:\cap\:\:\mathrm{A}\mathrm{E})\:}=\:\frac{\frac{{\mathrm{n}}_{\mathrm{A}\mathrm{B}1}}{{\mathrm{n}}_{\mathrm{A}\mathrm{B}+}\:+{\mathrm{n}}_{\mathrm{B}+}}}{\frac{{\mathrm{n}}_{\mathrm{A}\mathrm{B}1}+{\mathrm{n}}_{\mathrm{A}1}}{{\mathrm{n}}_{++}}}$$

with the terms of this equation computed as in Supplementary Table 1. A lift score > 1 indicates that the two events drug B and A ∩ AE are not independent, with higher values related to increased relevance of the interaction. Conviction evaluates whether the rule makes a wrong prediction based on the following formula:

Conviction (B → A ∩ AE) = $$\:\frac{1-\mathrm{S}\mathrm{u}\mathrm{p}\mathrm{p}\mathrm{o}\mathrm{r}\mathrm{t}\:\left(\mathrm{A}\:\cap\:\:\mathrm{A}\mathrm{E}\right)}{1-\:\mathrm{C}\mathrm{o}\mathrm{n}\mathrm{f}\mathrm{i}\mathrm{d}\mathrm{e}\mathrm{n}\mathrm{c}\mathrm{e}\:\left(\mathrm{B}\:\to\:\:\mathrm{A}\:\cap\:\:\mathrm{A}\mathrm{E}\right)}=\:\frac{\frac{1-({\mathrm{n}}_{\mathrm{A}\mathrm{B}1}+\:{\mathrm{n}}_{\mathrm{A}1})}{{\mathrm{n}}_{++}}}{\frac{1-\:{\mathrm{n}}_{\mathrm{A}\mathrm{B}1}}{\:{\mathrm{n}}_{\mathrm{A}\mathrm{B}+\:}+\:{\mathrm{n}}_{\mathrm{B}+}}}$$

Association rules were established as lift > 1 and conviction > 1, as in previous studies [25]. In addition, a sensitivity analysis was conducted adopting a stricter threshold of lift > 2 as this would correspond to a relative magnitude at least twice as high of the probability of observing A ∩ AE under the condition of drug B, compared to the probability of observing A ∩ AE [26].

Next, we evaluated the additive interaction (AI) and multiplicative interaction (MI) scores [25, 27]. AI is calculated as.

AI = risk difference _drug A drug B_ - (risk difference _drug A not drug B_ + risk difference _drug B not drug A_).

and an interaction under the additive assumption is established when the excess risk associated with the combination of drug A and drug B is higher than the sum of the risks associated with exposure to each drug in the absence of the other and therefore when AI > 0. MI is computed as the ratio between the risk ratio of the combination drug A and drug B and the product of the risk ratios associated with each drug separately, as in:

$$\:\mathrm{M}\mathrm{I}=\:\frac{{\mathrm{r}\mathrm{i}\mathrm{s}\mathrm{k}\:\mathrm{r}\mathrm{a}\mathrm{t}\mathrm{i}\mathrm{o}}_{\mathrm{d}\mathrm{r}\mathrm{u}\mathrm{g}\:\mathrm{A}\:\mathrm{d}\mathrm{r}\mathrm{u}\mathrm{g}\:\mathrm{B}\:}\:}{{\mathrm{r}\mathrm{i}\mathrm{s}\mathrm{k}\:\mathrm{r}\mathrm{a}\mathrm{t}\mathrm{i}\mathrm{o}}_{\mathrm{d}\mathrm{r}\mathrm{u}\mathrm{g}\:\mathrm{A}\:\mathrm{n}\mathrm{o}\mathrm{t}\:\mathrm{d}\mathrm{r}\mathrm{u}\mathrm{g}\:\mathrm{B}}\mathrm{*}\:{\mathrm{r}\mathrm{i}\mathrm{s}\mathrm{k}\:\mathrm{r}\mathrm{a}\mathrm{t}\mathrm{i}\mathrm{o}}_{\mathrm{d}\mathrm{r}\mathrm{u}\mathrm{g}\:\mathrm{B}\:\mathrm{n}\mathrm{o}\mathrm{t}\:\mathrm{d}\mathrm{r}\mathrm{u}\mathrm{g}\:\mathrm{A}}}$$

and an interaction on the multiplicative scale is established when MI > 1. Finally, we used the combination risk ratio (CRR), a detection algorithm based on the proportional reporting ratio (PRR) [28, 29] and computed as follows:

$$\:\mathrm{C}\mathrm{R}\mathrm{R}=\:\frac{{\mathrm{P}\mathrm{R}\mathrm{R}}_{\mathrm{D}\mathrm{r}\mathrm{u}\mathrm{g}\:\mathrm{A}\:\cap\:\:\mathrm{D}\mathrm{r}\mathrm{u}\mathrm{g}\:\mathrm{B}\:\:}\:\:}{\mathrm{m}\mathrm{a}\mathrm{x}\:({\mathrm{P}\mathrm{R}\mathrm{R}}_{\mathrm{D}\mathrm{r}\mathrm{u}\mathrm{g}\:\mathrm{A}\:},\:\:\:{\mathrm{P}\mathrm{R}\mathrm{R}}_{\mathrm{D}\mathrm{r}\mathrm{u}\mathrm{g}\:\:\mathrm{B}\:\:})}$$

A CRR > 2 is considered to be a signal of drug-drug interaction [29]. Analyses were conducted using R v. 4.4 [30].

## Results

### Description of esketamine-related AEs

Over the period 2014 Q3–2024 Q4, 14,198,145 AEs were reported in FAERS. After removal of duplicate reports and cases listed in the FAERS deleted files, 10,126,485 AEs were included in the analyses. A total of 393 cases in which esketamine was not listed as PS or SS, or indications were not related to psychiatric disorders were excluded, leading to 7,790 esketamine-related AE cases (7,711 cases with esketamine reported as PS, 79 as SS, for a total of 1,536 different PTs). These 7,790 cases (4,557 women [58.5%], 2,530 men [32.5%], 703 missing sex [9.0%]) were compared with 10,118,302 non-esketamine AE cases. Table 1 reports the main characteristics of the esketamine-related reports. The majority of AEs occurred in adults (median age: 46 years, interquartile range: 25 years), in the United States (6,060 cases, 77.8%) and were reported by healthcare professionals (35.6%) or physicians (32.1%).

**Table 1:** Characteristics of esketamine-related adverse events (*n* = 7,790 cases)

Variable	Value
Female, n (%)	4,557 (58.5%)
Male, n (%)	2,530 (32.5%)
Missing, n (%)	703 (9.0%)
Age at onset, median years (IQR)	48 (24)
Age at onset group	
Adults, n (%)	4,618 (59.3%)
Elderly, n (%)	748 (9.6%)
Neonates/infants, n (%)	5 (0.1%)
Adolescents, n (%)	55 (0.7%)
Missing, n (%)	2,364 (30.3%)
Country of occurrence	
US, n (%)	6,060 (77.8%)
Outside US, n (%)	1,624 (20.9%)
Missing, n (%)	106 (1.3%)
Reporting year	
2019, n (%)	563 (7.2%)
2020, n (%)	786 (10.1%)
2021, n (%)	966 (12.4%)
2022, n (%)	1,197 (15.4%)
2023, n (%)	1,726 (22.2%)
2024, n (%)	2,509 (32.2%)
Other/NA, n (%)	43 (0.5%)
Qualification of reporter	
Healthcare professionals	2,775 (35.6%)
Physicians	2,499 (32.1%)
Pharmacists	130 (1.7%)
Other health professionals	76 (1.0%)
Consumers	2,289 (29.4%)
Missing	21 (0.2%)

Reporting year was coded based on the event date or, if not available, the date in which the report was reported to FAERS. IQR, interquartile range.

### Identification of potential safety signals

Potential safety signals based on ROR were identified for 173 PTs with at least 5 reports. Table 2 provides disproportionality estimates for the top 30 PTs in terms of ROR values, while the complete list of PTs for which potential safety signals were identified is reported in Supplementary Table 2.

**Table 2:** Disproportionality estimates for the top 30 PTs based on ROR

AE	Esketamine (7,790 cases)	Non-esketamine (10,118,302 cases)	ROR (95% CI)
Dissociation	1,217 (15.6%)	1,713 (0.0%)	1,093.46 (1,012.06-1.06,181.41)
Sedation	829 (10.6%)	11,119 (0.1%)	108.25 (100.5–116.61.5.61)
Suicidal ideation	753 (9.7%)	41,145 (0.4%)	26.21 (24.3–28.27.3.27)
Nausea	483 (6.2%)	420,607 (4.2%)	1.52 (1.39–1.67)
Depression	448 (5.8%)	109,003 (1.1%)	5.6 (5.09–6.17)
Anxiety	410 (5.3%)	145,172 (1.4%)	3.82 (3.45–4.22)
Vomiting	395 (5.1%)	247,656 (2.4%)	2.13 (1.92–2.36)
Hypertension	379 (4.9%)	113,679 (1.1%)	4.5 (4.06–4.99)
Dizziness	350 (4.5%)	270,273 (2.7%)	1.71 (1.54–1.91)
Blood pressure increased	345 (4.4%)	87,692 (0.9%)	5.3 (4.76–5.91)
Hospitalisation	292 (3.7%)	74,056 (0.7%)	5.28 (4.7–5.94)
Product dose omission issue	258 (3.3%)	153,231 (1.5%)	2.23 (1.97–2.52)
Suicide attempt	248 (3.2%)	27,277 (0.3%)	12.16 (10.71–13.81)
Feeling abnormal	226 (2.9%)	132,892 (1.3%)	2.25 (1.97–2.56)
Completed suicide	168 (2.2%)	31,515 (0.3%)	7.05 (6.05–8.22)
Panic attack	163 (2.1%)	18,866 (0.2%)	11.44 (9.79–13.37)
Hallucination	157 (2%)	37,320 (0.4%)	5.56 (4.74–6.51)
Somnolence	123 (1.6%)	104,843 (1%)	1.53 (1.28–1.83)
Hallucination, visual	98 (1.3%)	10,251 (0.1%)	12.56 (10.28–15.35)
Device issue	96 (1.2%)	57,688 (0.6%)	2.18 (1.78–2.66)
Underdose	91 (1.2%)	45,000 (0.4%)	2.65 (2.15–3.25)
Seizure	88 (1.1%)	80,560 (0.8%)	1.42 (1.15–1.76)
Hypoaesthesia	86 (1.1%)	81,037 (0.8%)	1.38 (1.12–1.71)
Major depression	84 (1.1%)	3,758 (0%)	29.34 (23.61–36.46)
Surgery	82 (1.1%)	29,347 (0.3%)	3.66 (2.94–4.55)
Adverse event	81 (1%)	35,577 (0.4%)	2.98 (2.39–3.71)
Loss of consciousness	76 (1%)	65,154 (0.6%)	1.52 (1.21–1.91)
Euphoric mood	74 (0.9%)	5,087 (0.1%)	19.07 (15.14–24.01)
Crying	68 (0.9%)	18,601 (0.2%)	4.78 (3.76–6.07)
Agitation	65 (0.8%)	34,725 (0.3%)	2.44 (1.91–3.12)

Dissociation, sedation and suicidal ideation were among the top potential safety signals, in accordance with previous studies [7, 8, 13]. We identified novel potential signals for the PTs homicidal ideation (ROR = 5.30, 95% CI: 2.38–11.82) and substance use disorder (ROR = 6.12, 95% CI: 2.54–14.73). Other potential signals not identified in FAERS by previous studies, but already reported in the SPC, include the PTs aneurysm (ROR = 2.42, 95% CI, 1.01–5.82) and intracranial aneurysm (ROR = 3.46, 95% CI: 1.55–7.71), as well as the PTs seizure (ROR = 1.42, 95% CI: 1.15–1.76) and seizure like phenomena (ROR = 5.69, 95% CI: 2.37–13.71).

A longer TTO was observed in women (*n* = 1,625, median TTO: 56 days, IQR: 234 days) than men (*n* = 902, median TTO: 35 days, IQR: 166 days, Mann-Whitney U = 681,327, *p* = 0.003). While serious and non-serious cases did not differ for sex, age or weight (Table 3), a longer TTO was observed for serious compared with non-serious cases (60 vs. 3 days, *p* < 0.001, Table 3).

**Table 3:** Differences in demographic characteristics and frequency of AEs between serious and non-serious cases

	Non-serious cases (*n* = 1,527)	Serious cases (*n* = 7,760)	Statistics	*p*
Age, median years (IQR)^a^	46.5 (24)	48 (23)	2,047,059	0.08
Sex distribution				
Female, n (%)	1,009 (66.1%)	3,548 (63.8%)	2.68	0.10
Male, n (%)	518 (33.9%)	2,012 (36.2%)		
Weight, median Kg (IQR)^b^	77.8 (32)	79.1 (31.0)	133,901	0.89
Time-to-onset*,** median days (IQR)**^c^	**2 (40)**	**60 (227)**	**237**,**900**	**< 0.001**

Data available for ^a^ 926 not-serious and 4,590 serious cases; ^b^ 256 not-serious and 1,052 serious cases; ^c^ 375 not-serious and 2,224 serious cases. Abbreviations: IQR, interquartile range. Age, weight and time-to-onset were compared between serious and non-serious cases using Mann-Whitney U test, while sex and AEs with Pearson’s chi-squared test. Results in bold are statistically significant

The sensitivity analysis comparing esketamine to venlafaxine highlighted 72 PTs with potential safety signals (Supplementary Table 3). PTs with top signals in the main analysis such as dissociation, sedation and suicidal ideation were still significant in the sensitivity analysis, as were substance abuse (ROR = 2.46, 95% CI: 1.47–4.12) and seizure like phenomena (ROR = 3.03, 95% CI: 1.08–8.52), while other signals such as aneurysm and intracranial aneurysm were not identified.

**Fig. 1:**
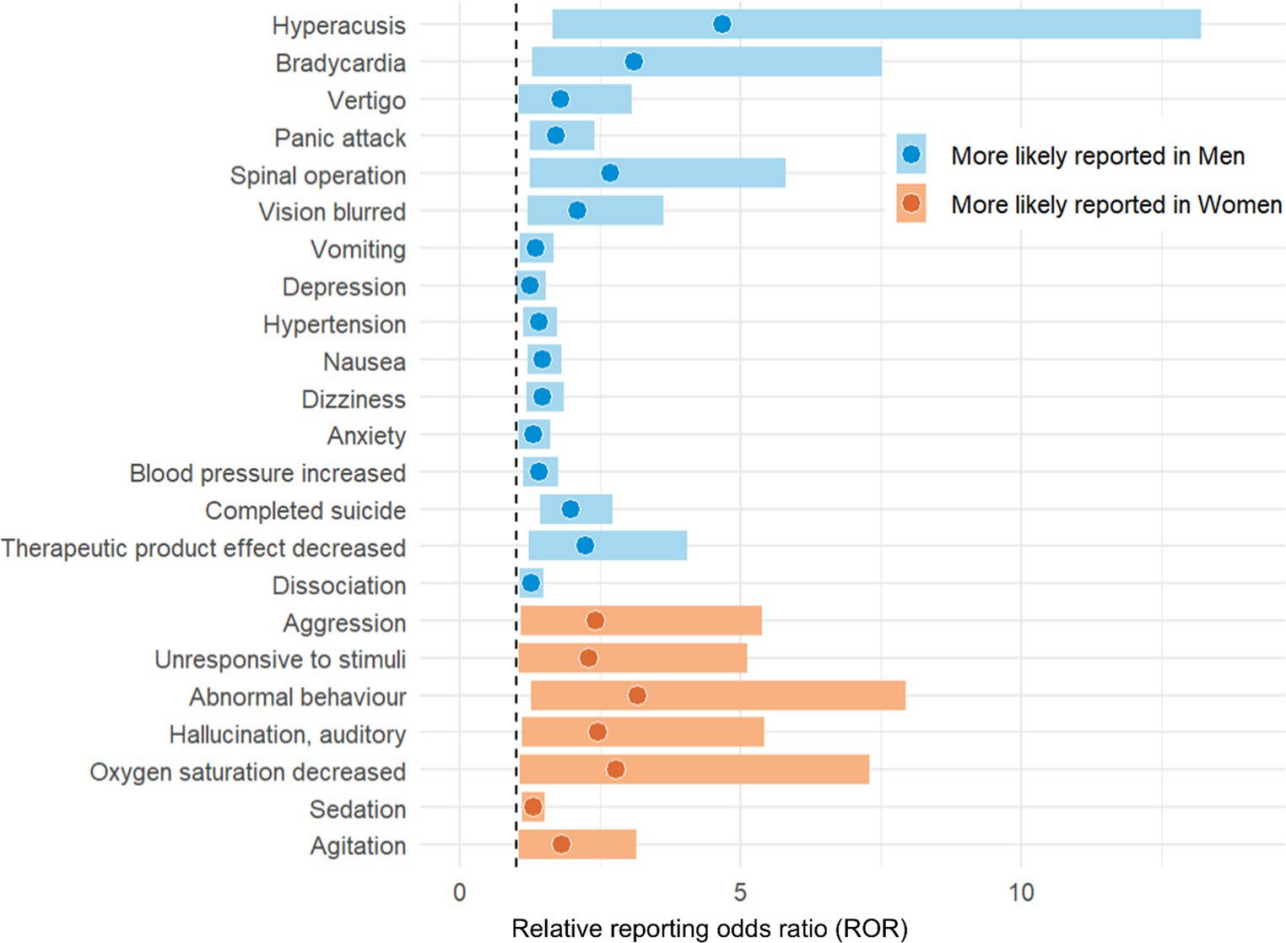
Forest plot showing AEs more likely to be reported in women or in men based on the relative reporting odds ratio (ROR)

We identified sex differences in potential safety signals for 23 PTs, seven of which were more likely to be reported in women while 16 in men (Fig. 1; Table 4). Among PTs more likely to be reported in women, four showed significant potential safety signals exclusively in women (oxygen saturation decreased, abnormal behaviour, unresponsive to stimuli and aggression), while three in both women and men (agitation, sedation and auditory hallucination, Table 4). Among PTs more likely to be reported in men, two showed potential safety signals exclusively in men (vision blurred and bradycardia), while all the others in both sexes (dissociation, therapeutic product effect decreased, completed suicide, blood pressure increased, anxiety, dizziness, nausea, hypertension, depression, vomiting, spinal operation, panic attack, vertigo and hyperacusis).

**Table 4:** Sex differences in potential safety signals

PT	*N*. reports females	ROR_females_ (95% CI)	*N*. reports males	ROR_males_ (95% CI)	Relative ROR 95% CI)
More likely to be reported in females					
Agitation	44	3.22 (2.39–4.33)	18	1.77 (1.12–2.82)	1.81 (1.04–3.15)
Sedation	520	135.82 (123.47–149.41.47.41)	294	104.46 (92.19–118.36.19.36)	1.30 (1.11–1.52)
Oxygen saturation decreased	25	1.58 (1.06–2.34)	5	0.56 (0.23–1.36)	2.79 (1.07–7.30)
Hallucination, auditory	27	8.44 (5.77–12.34)	8	3.44 (1.72–6.89)	2.45 (1.11–5.42)
Abnormal behaviour	19	3.22 (2.05–5.06)	6	1.02 (0.46–2.27)	3.17 (1.26–7.95)
Unresponsive to stimuli	25	4.62 (3.11–6.85)	8	2.01 (1.00–4.02.00.02)	2.30 (1.04–5.11)
Aggression	18	2.61 (1.64–4.15)	9	1.08 (0.56–2.08)	2.42 (1.08–5.39)
**More likely to be reported in males**					
Dissociation	759	1113.28 (1007.04–1230.72.04.72)	427	1405.53 (1228.92–1607.53.92.53)	1.26 (1.07–1.49)
Therapeutic product effect decreased	22	1.90 (1.25–2.89)	21	4.23 (2.75–6.50)	2.23 (1.22–4.06)
Completed suicide	66	4.82 (3.78–6.15)	85	9.48 (7.63–11.78)	1.97 (1.42–2.73)
Blood pressure increased	197	4.90 (4.25–5.65)	132	6.89 (5.78–8.21)	1.41 (1.12–1.76)
Anxiety	259	3.59 (3.17–4.07)	134	4.65 (3.91–5.54)	1.30 (1.05–1.61)
Dizziness	207	1.50 (1.30–1.72)	121	2.22 (1.85–2.66)	1.48 (1.18–1.86)
Nausea	311	1.35 (1.20–1.51)	141	2.00 (1.68–2.37)	1.48 (1.21–1.82)
Hypertension	214	4.10 (3.58–4.71)	145	5.75 (4.86–6.80)	1.40 (1.13–1.74)
Depression	282	5.23 (4.64–5.90)	143	6.52 (5.50–7.72)	1.25 (1.01–1.53)
Vomiting	253	1.99 (1.75–2.26)	123	2.66 (2.22–3.19)	1.34 (1.07–1.67)
Vision blurred	29	0.76 (0.53–1.09)	23	1.59 (1.05–2.40)	2.10 (1.21–3.63)
Spinal operation	12	2.87 (1.63–5.06)	14	7.70 (4.55–13.04)	2.68 (1.24–5.81)
Panic attack	100	9.91 (8.12–12.09)	58	17.02 (13.10–22.11.10.11)	1.72 (1.24–2.39)
Vertigo	39	2.02 (1.47–2.77)	21	3.62 (2.36–5.57)	1.80 (1.05–3.06)
Bradycardia	7	0.66 (0.32–1.39)	17	2.06 (1.28–3.32)	3.11 (1.29–7.51)
Hyperacusis	6	5.40 (2.42–12.05)	9	25.23 (13.04–48.83)	4.67 (1.65–13.21)

Relative ROR women (F/M) and men (M/F) are reported for AEs more likely to be reported in women or in men, respectively. CI, confidence interval; ROR, reporting odds ratio

A subgroup analysis comparing older patients with adults included 78 PTs for which at least 5 cases were reported in both age groups. We found 17 PTs to be more likely to be reported in older adults and 2 in adults (Table 5). Among those more likely to be reported in older adults, the majority were also significant in younger adults, while the three PTs drug ineffective, nervousness and migraine were only significant in older adults. Two PTs (hospitalization and hallucination) were more likely to be reported in younger adults, though they were also significant in older adults (Table 5).

**Table 5:** Age differences in potential safety signals

PT	*N*. reports older adults	ROR_older adults_ (95% CI)	*N*. reports adults	ROR_adults_ (95% CI)	Relative ROR 95% CI)
More likely to be reported in older adults					
Drug ineffective	62	1.69 (1.31–2.19)	248	0.92 (0.81–1.04)	1.85 (1.38–2.46)
Anxiety	43	6.58 (4.84–8.94)	283	3.43 (3.04–3.86)	1.92 (1.38–2.67)
Feeling abnormal	35	3.55 (2.53–4.99)	150	2.29 (1.94–2.69)	1.56 (1.07–2.26)
Sedation	114	198.48 (162.23–242.83.23.83)	697	138.40 (127.08–150.73.08.73)	1.43 (1.15–1.79)
Dissociation	166	3569.21 (2848.45–4472.35.45.35)	971	1054.53 (958.52–1160.16.52.16)	3.38 (2.65–4.32)
Depression	52	10.77 (8.13–14.25)	301	4.96 (4.42–5.58)	2.17 (1.60–2.94)
Suicide attempt	18	28.59 (17.89–45.69)	163	9.39 (8.02–10.98)	3.05 (1.86–4.99)
Suicidal ideation	53	49.26 (37.24–65.16)	570	25.28 (23.13–27.62)	1.95 (1.45–2.61)
Panic attack	15	22.25 (13.33–37.14)	118	9.91 (8.25–11.91)	2.25 (1.30–3.87)
Nervousness	6	2.46 (1.10–5.49)	10	0.80 (0.43–1.49)	3.08 (1.12–8.50)
Catatonia	5	30.39 (12.56–73.53)	7	4.44 (2.11–9.34)	6.85 (2.16–21.72)
Euphoric mood	7	42.56 (20.13–90.01)	48	16.79 (12.60–22.38.60.38)	2.53 (1.14–5.65)
Depressive symptom	5	55.23 (22.75–134.08.75.08)	7	6.13 (2.91–12.91)	9.00 (2.83–28.66)
Agitation	11	4.69 (2.59–8.50)	39	2.22 (1.62–3.04)	2.12 (1.08–4.15)
Diplopia	6	5.90 (2.64–13.18)	15	2.24 (1.35–3.72)	2.64 (1.02–6.82)
Dissociative disorder	10	1596.30 (740.13–3442.90.13.90)	38	158.48 (111.80–224.64.80.64)	10.07 (4.33–23.43)
Migraine	5	3.19 (1.32–7.68)	34	0.92 (0.66–1.29)	3.47 (1.35–8.89)
**More likely to be reported in adults**					
Hospitalisation	31	4.57 (3.20–6.55)	205	7.17 (6.23–8.25)	1.57 (1.07–2.30)
Hallucination	22	4.74 (3.11–7.24)	93	8.13 (6.61–9.99)	1.71 (1.07–2.74)

Relative ROR older adults (older adults/adults) and adults (adults/older adults) are reported for AEs more likely to be reported in older adults or in adults, respectively. Abbreviations: CI, confidence interval; ROR, reporting odds ratio

### Analysis of potential drug-drug interaction signals

Suggestive signals for potential drug-drug interactions based on all criteria were identified for four PTs (Table 6).

**Table 6:** Concomitant drugs with suggestive drug-drug interaction signals

Drug B	*N*. AE ESK	*N*. AE drug B	*N*. AE ESK and drug B	Lift	Conviction	AI	MI	CRR
Agitation								
Clonazepam	65	360	3	**5.27**	**1.00**	**0.14**	**12.23**	**17.95**
Bupropion	65	368	3	**6.68**	**1.00**	**0.10**	**7.26**	**13.81**
Blood pressure increased								
Alprazolam	345	168	3	0.89	1.00	0.14	15.55	3.97
Clonidine	345	307	3	**2.38**	**1.00**	**0.70**	**13.45**	**16.86**
Dissociation								
Alprazolam	1217	21	7	0.59	1.00	0.26	0.42	0.44
Clonazepam	1217	53	4	0.38	1.00	0.04	0.04	0.21
Lamotrigine	1217	24	4	0.58	1.00	0.02	0.07	0.19
Lorazepam	1217	27	4	0.35	1.00	0.08	0.13	0.25
Mirtazapine	1217	48	3	0.51	1.00	0.12	0.04	0.29
Pregabalin	1217	54	3	0.20	1.00	0.22	0.11	0.40
Quetiapine	1217	68	4	0.41	1.00	−0.04	0.02	0.12
Dizziness								
Venlafaxine	350	1011	3	**1.28**	**1.00**	**0.14**	**5.30**	**3.92**
Hypertension								
Duloxetine	379	393	3	0.80	1.00	0.11	6.99	3.23
Nausea								
Alprazolam	483	515	3	0.65	1.00	0.14	16.06	2.84
Quetiapine	483	732	3	0.80	1.00	0.05	4.48	1.38
Venlafaxine	483	1083	3	0.94	1.00	0.13	5.30	2.84
Sedation								
Alprazolam	829	167	5	0.62	1.00	0.19	1.23	2.49
Lorazepam	829	258	5	0.64	1.00	0.19	0.74	2.49
Vertigo								
Risperidone	64	76	3	**8.78**	**1.00**	**0.49**	**129.92**	**60.78**

In bold: concomitant drugs with significant drug-drug interaction signals based on all criteria. CRR, combination risk ratio; ESK, esketamine; AI, addictive interaction; MI, multiplicative interaction; N. AE, number of adverse events.

 Drugs with suggestive signals include serotonin and noradrenaline reuptake inhibitor antidepressants (venlafaxine), antidepressants with other mechanisms of action (bupropion), antipsychotics (risperidone), benzodiazepines (clonazepam) and antihypertensives (clonidine) (Table 6). For all these drugs we observed positive AI scores, suggestive of an interaction under the additive model, as well as MI scores > 1, suggestive of drug-drug interaction signals on the multiplicative scale. In addition, three out of the four potential drug-drug interaction signals remained significant in the sensitivity analysis considering a more restrictive threshold of lift > 2, while the potential signal for venlafaxine and dizziness was no longer significant.

## Discussion

In this study, we conducted an updated and comprehensive evaluation of AEs associated with esketamine in the FAERS spontaneous reporting system and, for the first time, evaluated drug-drug interaction signals to identify concomitant drugs that might exert an influence on esketamine-related AEs. We found that the number of esketamine reports has been steadily increasing from 2019 to 2024 and identified novel AEs, suggesting that post-marketing surveillance of this drug is still of high relevance. Among potential safety signals not identified by previous studies conducted in FAERS, some are included in the contraindications reported by the SPC (i.e. aneurysm or intracranial aneurysm), while others are reported in the SPC as rare events (such as seizures). Among novel AEs, we identified a safety signal for substance use disorder (ROR, 95% CI: 6.12, 2.54–14.73). In accordance, we confirmed potential signals for alcohol abuse and alcohol poisoning reported by a recent study conducted in FAERS [13] but not by an analysis conducted in the World Health Organization pharmacovigilance database [10]. These contrasting results call for further analyses on the potential risk of substance use disorders related to treatment with esketamine.

A large number of potential safety signals were confirmed in the sensitivity analysis in which esketamine was compared with venlafaxine, supporting the robustness of results for AEs such as dissociation, sedation, substance abuse, suicidal ideation, self-injurious ideation, suicide attempt and completed suicide, as well as for neurological potential safety signals such as seizure like phenomena or urological AEs such as cystitis interstitial (Supplementary Table 3).

In analyses stratified based on seriousness, we found that serious AEs were associated with a longer TTO compared with non-serious AEs (median days = 60 vs. 2, *p* < 0.001). While analyses of clinical trials did not identify new potential safety signals during long-term treatment (up to 4.5 years) with intermittent-dosed esketamine plus oral antidepressants [31], our result supports the need to conduct a careful evaluation of the risk-benefit ratio for long-term treatment.

The majority of reports involved female patients (58.5%), in accordance with previous studies [7, 8], but this higher percentage might be explained by a number of factors including the higher prevalence of MDD in women, and therefore potential differences in the rates of drug use, the fact that women generally show more frequent and serious AEs compared with men [32], as well as potential reporting bias, since previous studies suggested that women have a higher likelihood of reporting an ADR than men [33].

While the analysis of spontaneous reporting data does not allow to draw any conclusion on differences in the frequency of AEs, our results point to relevant sex differences for some characteristics of the esketamine potential safety signals. We observed a longer TTO in women compared with men (Mann-Whitney U = 681,327, *p* = 0.003), with no significant difference in the frequency of serious cases. In addition, while a recent study focused on sex differences only identified few PTs with higher risk in men (completed suicide, decreased therapeutic product effect, urinary retention and hypertension) [13], our analyses identified sex differences in potential safety signals for 23 PTs, seven of which showed higher risk in women while 16 in men. We confirmed higher risk in men for three of the four PTs identified by the previous study (completed suicide, decreased therapeutic effect, hypertension, but not urinary retention) and identified, for the first time, 13 other PTs with higher risk in men (Table 4). Among these, vision blurred and bradycardia did not show safety signals in women, while the other showed potential safety signals in both sexes (through they are more likely to be reported in men). In addition, we identified four PTs showing significant potential safety signals exclusively in women (oxygen saturation decreased, abnormal behaviour, unresponsive to stimuli and aggression), and three other PTs showing potential safety signals in both sexes but that are more likely to be reported in women (agitation, sedation and auditory hallucination). While the majority of PTs did not show sex differences, our results pointing to gender differences in TTO as well as in several PTs, highlight the need to further study gender differences in the occurrence of esketamine-related AEs. While age was not significantly associated with AE seriousness, we identified 17 PTs more likely to be reported in older adults compared with adults, while only two were more likely to be reported in younger compared with older adults. Drug ineffective was one of the PTs found to be more likely reported in older adults. While this finding suggests that older patients might more often experience a lack of response to esketamine, a previous post-hoc analysis of the SUSTAIN-2 phase 3 study suggested comparable efficacy outcomes between younger and older patients with TRD [34]. Nonetheless, our observation of a high number of potential safety signals found to be impacted by age supports the need for further studies focused on older patients with TRD treated with esketamine.

While clinical trials are typically designed to establish the efficacy and safety of a single drug, investigating drug-drug interaction signals is highly important considering the high rates of polypharmacy in real-world settings. Known interactions of esketamine include central nervous system depressants that may increase sedation (e.g. benzodiazepines, opioids, alcohol), and drugs that may increase blood pressure such as psychostimulants (e.g., amphetamines, methylphenidate, modafinil, armodafinil), xanthine derivatives, ergometrine, thyroid hormones, vasopressin, or monoamine oxidase inhibitors [14]. In addition, potential pharmacokinetics interactions have been suggested. The cytochrome P450 (CYP450) enzymes CYP2B6 and CYP3A4 are the main enzymes responsible for N-demethylation of esketamine to noresketamine, with other enzymes such as CYP2C19 and CYP2C9 contributing to a much smaller extent. While potential drug-drug interactions between esketamine and CYP2B6 (e.g. ticlopidine) or CYP3A4 (e.g. clarithromycin) inhibitors have been suggested [4, 14], we found no significant signals for these drugs in our analysis. However, we identified potential drug-drug interaction signals between esketamine and drugs metabolized by CYP3A4 (clonazepam and venlafaxine) and CYP2B6 (bupropion). Among the most interesting results, risperidone showed the strongest potential signals for drug-drug interaction with esketamine for the “vertigo” PT. This potential signal remained significant when considering a more restrictive threshold of lift > 2. Impairment of postural stability is more frequent with typical antipsychotic drugs, though risperidone has been previously suggested to alter balance control [35]. While no study investigated potential interactions between risperidone and esketamine, a previous systematic review including three neuroimaging studies suggested an attenuating effect of risperidone on ketamine-induced brain perfusion changes [36]. The strong signal for drug-drug interaction with esketamine on a multiplicative scale observed in our data support the need of studies evaluating the risks associated with esketamine combinations in real-world settings.

Our results have to be interpreted in light of some limitations. Spontaneous reporting systems are an important source of data for post-marketing surveillance of drug safety, as they allow to detect safety signals related to rare but serious AEs, populations excluded from clinical trials or drug-drug interactions [37]. However, they have relevant limitations such as the lack of a denominator for the number of exposed to a drug without AEs, variable quality of the reported data and under-reporting [38]. As in previous studies, some of the detected potential safety signals were related to esketamine indications (e.g., “depression”, “suicide ideation” or “suicide attempt”). Therefore, it is not clear whether these suspect AEs are part of the underlying disorder, are related to lack of efficacy or esketamine or might be induced by esketamine. In addition, while we excluded from the drug-drug interaction analysis reports in which drugs were only reported as “concomitant”, some of the potential drug-drug interaction signals might be explained by the fact that the analyzed drug has an indication for the observed AE (e.g. clonidine for the AE “hypertension”). Comorbid disorders such as liver or renal disorders, as well as genetic variants able to impact pharmacokinetics or pharmacodynamics, are not consistently reported in FAERS and might affect the risk to develop AEs. While an analysis of these factors could not be conducted in the present study, studies to be conducted in clinical cohorts with a deep phenotyping would be important to address the role of these factors. Finally, while we observed serious AEs to be characterized by a longer TTO compared with non-serious AEs, it must be underlined that serious cases are more likely to be reported and that it cannot be excluded that a longer observation period might result in more ADRs, including more serious ones, being observed.

In conclusion, this study provides a comprehensive and updated picture of potential signals of AEs associated with esketamine based on a comprehensive analysis of the FAERS database. We observed a higher TTO in serious compared to non-serious AEs, while our results do not support an effect of age and sex as risk factors related to AE seriousness. In addition, our results point to sex differences for some of the identified AEs and identified some concomitant drugs as factors able to increase the risk of esketamine-related AEs. While the identification of potential signals in FAERS does not establish causal relationships but only points to statistical associations warranting further investigation, our results support the importance of a careful evaluation of the risk-benefit ratio especially in patients treated with multiple drugs.

## Supplementary Information

Below is the link to the electronic supplementary material.

Supplementary Material 1

## Data Availability

All data used in this study are publicly available and key scripts are available at https://github.com/claudiapis/faers\_esketamine.
