# Correction to: A real-world pharmacovigilance study of adverse events associated with esketamine: disproportionality analysis and detection of potential drug-drug interaction signals

**DOI:** 10.1007/s00228-026-04007-9

**Published:** 2026-02-23

**Authors:** Claudia Pisanu, Shungo Imai, Masami Tsuchiya, Mari Inoue, Keisuke Ikegami, Gianpaolo Zammarchi, Hayato Kizaki, Satoko Hori

**Affiliations:** 1 Department of Biomedical Sciences, Section of Neuroscience and Clinical Pharmacology, University of Cagliari, Cagliari, Italy; 2 Division of Drug Informatics, Keio University Faculty of Pharmacy, Tokyo, Japan; 3 Department of Economics and Business Science, University of Cagliari, Cagliari, Italy

**Correction to: European Journal of Clinical Pharmacology (2026) 82:13** 10.1007/s00228-025-03954-z

At page 13, the correct version of “ 60 vs. 3 days, p < 0.001, Table 3” is “60 vs. 2 days, p < 0.001, Table 3).

Corrected Tables 2, 3, 4 and 5 are shown in this paper.

**Table 2:** Disproportionality estimates for the top 30 PTs based on ROR

AE	Esketamine (7,790 cases)	Non-esketamine (10,118,302 cases)	ROR (95% CI)
Dissociation	1,217 (15.6%)	1,713 (0.0%)	1,093.46 (1,012.06-1,181.41)
Sedation	829 (10.6%)	11,119 (0.1%)	108.25 (100.50–116.61)
Suicidal ideation	753 (9.7%)	41,145 (0.4%)	26.21 (24.30–28.27)
Nausea	483 (6.2%)	420,607 (4.2%)	1.52 (1.39–1.67)
Depression	448 (5.8%)	109,003 (1.1%)	5.60 (5.09–6.17)
Anxiety	410 (5.3%)	145,172 (1.4%)	3.82 (3.45–4.22)
Vomiting	395 (5.1%)	247,656 (2.4%)	2.13 (1.92–2.36)
Hypertension	379 (4.9%)	113,679 (1.1%)	4.05 (4.06–4.99)
Dizziness	350 (4.5%)	270,273 (2.7%)	1.71 (1.54–1.91)
Blood pressure increased	345 (4.4%)	87,692 (0.9%)	5.30 (4.76–5.91)
Hospitalisation	292 (3.7%)	74,056 (0.7%)	5.28 (4.7–5.94)
Product dose omission issue	258 (3.3%)	153,231 (1.5%)	2.23 (1.97–2.52)
Suicide attempt	248 (3.2%)	27,277 (0.3%)	12.16 (10.71–13.81)
Feeling abnormal	226 (2.9%)	132,892 (1.3%)	2.25 (1.97–2.56)
Completed suicide	168 (2.2%)	31,515 (0.3%)	7.05 (6.05–8.22)
Panic attack	163 (2.1%)	18,866 (0.2%)	11.44 (9.79–13.37)
Hallucination	157 (2%)	37,320 (0.4%)	5.56 (4.74–6.51)
Somnolence	123 (1.6%)	104,843 (1%)	1.53 (1.28–1.83)
Hallucination, visual	98 (1.3%)	10,251 (0.1%)	12.56 (10.28–15.35)
Device issue	96 (1.2%)	57,688 (0.6%)	2.18 (1.78–2.66)
Underdose	91 (1.2%)	45,000 (0.4%)	2.65 (2.15–3.25)
Seizure	88 (1.1%)	80,560 (0.8%)	1.42 (1.15–1.76)
Hypoaesthesia	86 (1.1%)	81,037 (0.8%)	1.38 (1.12–1.71)
Major depression	84 (1.1%)	3,758 (0%)	29.34 (23.61–36.46)
Surgery	82 (1.1%)	29,347 (0.3%)	3.66 (2.94–4.55)
Adverse event	81 (1%)	35,577 (0.4%)	2.98 (2.39–3.71)
Loss of consciousness	76 (1%)	65,154 (0.6%)	1.52 (1.21–1.91)
Euphoric mood	74 (0.9%)	5,087 (0.1%)	19.07 (15.14–24.01)
Crying	68 (0.9%)	18,601 (0.2%)	4.78 (3.76–6.07)
Agitation	65 (0.8%)	34,725 (0.3%)	2.44 (1.91–3.12)

**Table 3:** Differences in demographic characteristics and frequency of AEs between serious and non-serious cases

	Non-serious cases (n = 1,527)	Serious cases (n = 5,560)	Statistics	p
Age, median years (IQR)^a^	46.5 (24)	48 (23)	2,047,059	0.08
Sex distribution				
Female, n (%)	1,009 (66.1%)	3,548 (63.8%)	2.68	0.10
Male, n (%)	518 (33.9%)	2,012 (36.2%)		
Weight, median Kg (IQR)^b^	77.8 (32)	79.1 (31.0)	133,901	0.89
Time-to-onset*, median days (IQR) ^c^	**2 (40)**	**60 (227)**	**237,900**	**< 0.001**

Data available for ^a ^926 not-serious and 4,590 serious cases; ^b^ 256 not-serious and 1,052 serious cases; ^c^ 375 not-serious and 2,224 serious cases. Abbreviations: IQR, interquartile range. Age, weight and time-to-onset were compared between serious and non-serious cases using Mann-Whitney U test, while sex and AEs with Pearson’s chi-squared test. Results in bold are statistically significant

**Table 4:** Sex differences in potential safety signals

PT	N. reports females	ROR_females_ (95% CI)	N. reports males	ROR_males_ (95% CI)	Relative ROR 95% CI)
**More likely to be reported in females**					
Agitation	44	3.22 (2.39–4.33)	18	1.77 (1.12–2.82)	1.81 (1.04–3.15)
Sedation	520	135.82 (123.47–149.41)	294	104.46 (92.19–118.36)	1.30 (1.11–1.52)
Oxygen saturation decreased	25	1.58 (1.06–2.34)	5	0.56 (0.23–1.36)	2.79 (1.07–7.30)
Hallucination, auditory	27	8.44 (5.77–12.34)	8	3.44 (1.72–6.89)	2.45 (1.11–5.42)
Abnormal behaviour	19	3.22 (2.05–5.06)	6	1.02 (0.46–2.27)	3.17 (1.26–7.95)
Unresponsive to stimuli	25	4.62 (3.11–6.85)	8	2.01 (1.00–4.02)	2.30 (1.04–5.11)
Aggression	18	2.61 (1.64–4.15)	9	1.08 (0.56–2.08)	2.42 (1.08–5.39)
**More likely to be reported in males**					
Dissociation	759	1,113.28 (1,007.04–1,230.72)	427	1,405.53 (1,228.92–1,607.53)	1.26 (1.07–1.49)
Therapeutic product effect decreased	22	1.90 (1.25–2.89)	21	4.23 (2.75–6.50)	2.23 (1.22–4.06)
Completed suicide	66	4.82 (3.78–6.15)	85	9.48 (7.63–11.78)	1.97 (1.42–2.73)
Blood pressure increased	197	4.90 (4.25–5.65)	132	6.89 (5.78–8.21)	1.41 (1.12–1.76)
Anxiety	259	3.59 (3.17–4.07)	134	4.65 (3.91–5.54)	1.30 (1.05–1.61)
Dizziness	207	1.50 (1.30–1.72)	121	2.22 (1.85–2.66)	1.48 (1.18–1.86)
Nausea	311	1.35 (1.20–1.51)	141	2.00 (1.68–2.37)	1.48 (1.21–1.82)
Hypertension	214	4.10 (3.58–4.71)	145	5.75 (4.86–6.80)	1.40 (1.13–1.74)
Depression	282	5.23 (4.64–5.90)	143	6.52 (5.50–7.72)	1.25 (1.01–1.53)
Vomiting	253	1.99 (1.75–2.26)	123	2.66 (2.22–3.19)	1.34 (1.07–1.67)
Vision blurred	29	0.76 (0.53–1.09)	23	1.59 (1.05–2.40)	2.10 (1.21–3.63)
Spinal operation	12	2.87 (1.63–5.06)	14	7.70 (4.55–13.04)	2.68 (1.24–5.81)
Panic attack	100	9.91 (8.12–12.09)	58	17.02 (13.10–22.11)	1.72 (1.24–2.39)
Vertigo	39	2.02 (1.47–2.77)	21	3.62 (2.36–5.57)	1.80 (1.05–3.06)
Bradycardia	7	0.66 (0.32–1.39)	17	2.06 (1.28–3.32)	3.11 (1.29–7.51)
Hyperacusis	6	5.40 (2.42–12.05)	9	25.23 (13.04–48.83)	4.67 (1.65–13.21)

Relative ROR women (F/M) and men (M/F) are reported for AEs more likely to be reported in women or in men, respectively. Abbreviations: CI, confidence interval; ROR, reporting odds ratio

**Table 5:** Age differences in potential safety signals

PT	N. reports older adults	ROR_older adults_ (95% CI)	N. reports adults	ROR_adults_ (95% CI)	Relative ROR (95% CI)
**More likely to be reported in older adults**					
Drug ineffective	62	1.69 (1.31–2.19)	248	0.92 (0.81–1.04)	1.85 (1.38–2.46)
Anxiety	43	6.58 (4.84–8.94)	283	3.43 (3.04–3.86)	1.92 (1.38–2.67)
Feeling abnormal	35	3.55 (2.53–4.99)	150	2.29 (1.94–2.69)	1.56 (1.07–2.26)
Sedation	114	198.48 (162.23–242.83)	697	138.40 (127.08–150.73)	1.43 (1.15–1.79)
Dissociation	166	3,569.21 (2,848.45–4,472.35)	971	1,054.53 (958.52–1,160.16)	3.38 (2.65–4.32)
Depression	52	10.77 (8.13–14.25)	301	4.96 (4.42–5.58)	2.17 (1.60–2.94)
Suicide attempt	18	28.59 (17.89–45.69)	163	9.39 (8.02–10.98)	3.05 (1.86–4.99)
Suicidal ideation	53	49.26 (37.24–65.16)	570	25.28 (23.13–27.62)	1.95 (1.45–2.61)
Panic attack	15	22.25 (13.33–37.14)	118	9.91 (8.25–11.91)	2.25 (1.30–3.87)
Nervousness	6	2.46 (1.10–5.49)	10	0.80 (0.43–1.49)	3.08 (1.12–8.50)
Catatonia	5	30.39 (12.56–73.53)	7	4.44 (2.11–9.34)	6.85 (2.16–21.72)
Euphoric mood	7	42.56 (20.13–90.01)	48	16.79 (12.60–22.38)	2.53 (1.14–5.65)
Depressive symptom	5	55.23 (22.75–134.08)	7	6.13 (2.91–12.91)	9.00 (2.83–28.66)
Agitation	11	4.69 (2.59–8.50)	39	2.22 (1.62–3.04)	2.12 (1.08–4.15)
Diplopia	6	5.90 (2.64–13.18)	15	2.24 (1.35–3.72)	2.64 (1.02–6.82)
Dissociative disorder	10	1,596.30 (740.13–3,442.90)	38	158.48 (111.80–224.64)	10.07 (4.33–23.43)
Migraine	5	3.19 (1.32–7.68)	34	0.92 (0.66–1.29)	3.47 (1.35–8.89)
**More likely to be reported in adults**					
Hospitalisation	31	4.57 (3.20–6.55)	205	7.17 (6.23–8.25)	1.57 (1.07–2.30)
Hallucination	22	4.74 (3.11–7.24)	93	8.13 (6.61–9.99)	1.71 (1.07–2.74)

Relative ROR older adults (older adults/adults) and adults (adults/older adults) are reported for AEs more likely to be reported in older adults or in adults, respectively. Abbreviations: CI, confidence interval; ROR, reporting odds ratio

