# Supplementary material for: A real-world pharmacovigilance study of adverse events associated with esketamine: disproportionality analysis and detection of potential drug-drug interaction signals
Source: Eur J Clin Pharmacol. 2025 Dec 22;82(1):13. doi: 10.1007/s00228-025-03954-z (PMC12722400; doi:10.1007/s00228-025-03954-z)
Supplement: Supplementary file 1 — Supplementary Material 1 [file 228_2025_3954_MOESM1_ESM.docx]

**Supplementary tables**

**Table S1. Contingency table for the association rule mining**

|  | **Suspected AE** | **All other AEs** | **Total** |
| --- | --- | --- | --- |
| **Drug A and Drug B** | n_AB1_ | n_AB2_ | n_AB+_ |
| **Drug A, not Drug B** | n_A1_ | n_A2_ | n_A+_ |
| **Drug B, not Drug A** | n_B1_ | n_B2_ | n_B+_ |
| **All other drugs** | n_O1_ | n_O2_ | n_O+_ |
| **Total** | n_+1_ | n_+2_ | n_++_ |

**Table S2. Disproportionality estimates for 173 AEs with potential safety signals**

| **PT** | **Esketamine (7,790 cases)** | **Non-esketamine (10,118,302 cases)** | **ROR (95% CI)** |
| --- | --- | --- | --- |
| Dissociation | 1,217 (15.6%) | 1,713 (0.0%) | 1,093.46 (1,012.06-1,181.41) |
| Sedation | 829 (10.6%) | 11,119 (0.1%) | 108.25 (100.50-116.61) |
| Suicidal ideation | 753 (9.7%) | 41,145 (0.4%) | 26.21 (24.30-28.27) |
| Nausea | 483 (6.2%) | 420,607 (4.2%) | 1.52 (1.39-1.67) |
| Depression | 448 (5.8%) | 109,003 (1.1%) | 5.60 (5.09-6.17) |
| Anxiety | 410 (5.3%) | 145,172 (1.4%) | 3.82 (3.45-4.22) |
| Vomiting | 395 (5.1%) | 247,656 (2.4%) | 2.13 (1.92-2.36) |
| Hypertension | 379 (4.9%) | 113,679 (1.1%) | 4.50 (4.06-4.99) |
| Dizziness | 350 (4.5%) | 270,273 (2.7%) | 1.71 (1.54-1.91) |
| Blood pressure increased | 345 (4.4%) | 87,692 (0.9%) | 5.30 (4.76-5.91) |
| Hospitalisation | 292 (3.7%) | 74,056 (0.7%) | 5.28 (4.70-5.94) |
| Product dose omission issue | 258 (3.3%) | 153,231 (1.5%) | 2.23 (1.97-2.52) |
| Suicide attempt | 248 (3.2%) | 27,277 (0.3%) | 12.16 (10.71-13.81) |
| Feeling abnormal | 226 (2.9%) | 132,892 (1.3%) | 2.25 (1.97-2.56) |
| Completed suicide | 168 (2.2%) | 31,515 (0.3%) | 7.05 (6.05-8.22) |
| Panic attack | 163 (2.1%) | 18,866 (0.2%) | 11.44 (9.79-13.37) |
| Hallucination | 157 (2.0%) | 37,320 (0.4%) | 5.56 (4.74-6.51) |
| Somnolence | 123 (1.6%) | 104,843 (1%) | 1.53 (1.28-1.83) |
| Hallucination, visual | 98 (1.3%) | 10,251 (0.1%) | 12.56 (10.28-15.35) |
| Device issue | 96 (1.2%) | 57,688 (0.6%) | 2.18 (1.78-2.66) |
| Underdose | 91 (1.2%) | 45,000 (0.4%) | 2.65 (2.15-3.25) |
| Seizure | 88 (1.1%) | 80,560 (0.8%) | 1.42 (1.15-1.76) |
| Hypoaesthesia | 86 (1.1%) | 81,037 (0.8%) | 1.38 (1.12-1.71) |
| Major depression | 84 (1.1%) | 3,758 (0.0%) | 29.34 (23.61-36.46) |
| Surgery | 82 (1.1%) | 29,347 (0.3%) | 3.66 (2.94-4.55) |
| Adverse event | 81 (1.0%) | 35,577 (0.4%) | 2.98 (2.39-3.71) |
| Loss of consciousness | 76 (1.0%) | 65,154 (0.6%) | 1.52 (1.21-1.91) |
| Euphoric mood | 74 (0.9%) | 5,087 (0.1%) | 19.07 (15.14-24.01) |
| Crying | 68 (0.9%) | 18,601 (0.2%) | 4.78 (3.76-6.07) |
| Agitation | 65 (0.8%) | 34,725 (0.3%) | 2.44 (1.91-3.12) |
| Device malfunction | 65 (0.8%) | 35,751 (0.4%) | 2.37 (1.86-3.03) |
| Vertigo | 64 (0.8%) | 34,242 (0.3%) | 2.44 (1.91-3.12) |
| Dysgeusia | 61 (0.8%) | 39,462 (0.4%) | 2.02 (1.57-2.59) |
| Dissociative disorder | 59 (0.8%) | 316 (0.0%) | 244.36 (184.89-322.95) |
| Treatment noncompliance | 57 (0.7%) | 28,361 (0.3%) | 2.62 (2.02-3.40) |
| Depressed mood | 55 (0.7%) | 30,051 (0.3%) | 2.39 (1.83-3.11) |
| Intentional self-injury | 53 (0.7%) | 11,381 (0.1%) | 6.08 (4.64-7.98) |
| Therapeutic product effect decreased | 48 (0.6%) | 24,264 (0.2%) | 2.58 (1.94-3.43) |
| Incorrect dose administered by device | 48 (0.6%) | 15452 (0.2%) | 4.05 (3.05-5.39) |
| Mental disorder | 46 (0.6%) | 21463 (0.2%) | 2.79 (2.09-3.74) |
| Therapeutic response decreased | 43 (0.6%) | 27530 (0.3%) | 2.03 (1.51-2.75) |
| Exposure during pregnancy | 39 (0.5%) | 33531 (0.3%) | 1.51 (1.10-2.07) |
| Mental impairment | 39 (0.5%) | 12507 (0.1%) | 4.07 (2.97-5.57) |
| Taste disorder | 38 (0.5%) | 13,569 (0.1%) | 3.65 (2.65-5.02) |
| Hallucination, auditory | 36 (0.5%) | 7,974 (0.1%) | 5.89 (4.24-8.17) |
| Feeling drunk | 34 (0.4%) | 4,065 (0.0%) | 10.91 (7.78-15.3) |
| Unresponsive to stimuli | 34 (0.4%) | 13,416 (0.1%) | 3.30 (2.36-4.63) |
| Psychotic disorder | 34 (0.4%) | 13,025 (0.1%) | 3.40 (2.43-4.77) |
| Mania | 34 (0.4%) | 7,514 (0.1%) | 5.90 (4.21-8.27) |
| Dysarthria | 34 (0.4%) | 18,995 (0.2%) | 2.33 (1.66-3.27) |
| Neoplasm malignant | 33 (0.4%) | 28,778 (0.3%) | 1.49 (1.06-2.10) |
| Fear | 33 (0.4%) | 11,959 (0.1%) | 3.60 (2.55-5.06) |
| Sensory disturbance | 33 (0.4%) | 8,080 (0.1%) | 5.32 (3.78-7.50) |
| Road traffic accident | 32 (0.4%) | 21,785 (0.2%) | 1.91 (1.35-2.71) |
| Speech disorder | 31 (0.4%) | 28,029 (0.3%) | 1.44 (1.01-2.05) |
| Emotional disorder | 30 (0.4%) | 14,853 (0.1%) | 2.63 (1.84-3.77) |
| Anger | 30 (0.4%) | 17,154 (0.2%) | 2.28 (1.59-3.26) |
| Withdrawal syndrome | 29 (0.4%) | 20,640 (0.2%) | 1.83 (1.27-2.63) |
| Depressed level of consciousness | 29 (0.4%) | 18,363 (0.2%) | 2.06 (1.43-2.96) |
| Aggression | 28 (0.4%) | 22,551 (0.2%) | 1.61 (1.11-2.34) |
| Disorientation | 27 (0.3%) | 19,456 (0.2%) | 1.81 (1.24-2.63) |
| Nasal discomfort | 27 (0.3%) | 4,401 (0.0%) | 7.99 (5.47-11.68) |
| Spinal operation | 26 (0.3%) | 8,093 (0.1%) | 4.18 (2.84-6.15) |
| Abnormal behaviour | 26 (0.3%) | 17,228 (0.2%) | 1.96 (1.34-2.89) |
| Post-traumatic stress disorder | 25 (0.3%) | 3,876 (0.0%) | 8.40 (5.67-12.46) |
| Hypersomnia | 25 (0.3%) | 16,698 (0.2%) | 1.95 (1.31-2.89) |
| Bipolar disorder | 25 (0.3%) | 5,169 (0.1%) | 6.30 (4.25-9.34) |
| Thinking abnormal | 25 (0.3%) | 9,447 (0.1%) | 3.45 (2.33-5.10) |
| Diplopia | 24 (0.3%) | 13,312 (0.1%) | 2.35 (1.57-3.50) |
| Derealisation | 24 (0.3%) | 1,034 (0.0%) | 30.24 (20.16-45.35) |
| Hypoaesthesia oral | 24 (0.3%) | 8,315 (0.1%) | 3.76 (2.52-5.61) |
| Mood altered | 24 (0.3%) | 14,180 (0.1%) | 2.20 (1.47-3.29) |
| Paranoia | 23 (0.3%) | 7,948 (0.1%) | 3.77 (2.50-5.68) |
| Restlessness | 23 (0.3%) | 19,517 (0.2%) | 1.53 (1.02-2.31) |
| Nightmare | 23 (0.3%) | 16,113 (0.2%) | 1.86 (1.23-2.80) |
| Eating disorder | 22 (0.3%) | 13,324 (0.1%) | 2.15 (1.41-3.27) |
| Self-injurious ideation | 22 (0.3%) | 1,933 (0.0%) | 14.82 (9.73-22.58) |
| Product administration error | 22 (0.3%) | 13,849 (0.1%) | 2.07 (1.36-3.14) |
| Morbid thoughts | 21 (0.3%) | 504 (0.0%) | 54.26 (35.05-84.01) |
| Altered state of consciousness | 20 (0.3%) | 12,029 (0.1%) | 2.16 (1.39-3.36) |
| Substance abuse | 19 (0.2%) | 4,243 (0.0%) | 5.83 (3.71-9.15) |
| Depressive symptom | 19 (0.2%) | 1,921 (0.0%) | 12.88 (8.19-20.24) |
| Paralysis | 19 (0.2%) | 7,690 (0.1%) | 3.21 (2.05-5.05) |
| Micturition urgency | 19 (0.2%) | 6,574 (0.1%) | 3.76 (2.04-5.90) |
| Product complaint | 18 (0.2%) | 11,873 (0.1%) | 1.97 (1.24-3.13) |
| Hypertensive crisis | 18 (0.2%) | 6,156 (0.1%) | 3.80 (2.39-6.05) |
| Product delivery mechanism issue | 17 (0.2%) | 2,416 (0.0%) | 9.16 (5.68-14.76) |
| Negative thoughts | 17 (0.2%) | 1,596 (0.0%) | 13.86 (8.59-22.37) |
| Psychogenic seizure | 17 (0.2%) | 560 (0.0%) | 39.51 (24.38-64.05) |
| Hypokinesia | 17 (0.2%) | 8791 (0.1%) | 2.52 (1.56-4.05) |
| Panic disorder | 17 (0.2%) | 2,101 (0.0%) | 10.53 (6.53-16.98) |
| Autoscopy | 16 (0.2%) | 370 (0.0%) | 56.28 (34.1-92.88) |
| Akathisia | 16 (0.2%) | 5,628 (0.1%) | 3.70 (2.26-6.04) |
| Device defective | 16 (0.2%) | 7,529 (0.1%) | 2.76 (1.69-4.52) |
| Illusion | 16 (0.2%) | 854 (0.0%) | 24.38 (14.86-40) |
| Hyperacusis | 15 (0.2%) | 2,014 (0.0%) | 9.69 (5.83-16.11) |
| Rehabilitation therapy | 15 (0.2%) | 2,702 (0.0%) | 7.22 (4.35-12.00) |
| Drug delivery system malfunction | 15 (0.2%) | 2,816 (0.0%) | 6.93 (4.17-11.52) |
| Concussion | 15 (0.2%) | 4,889 (0.0%) | 3.99 (2.40-6.63) |
| Feeling of despair | 15 (0.2%) | 2,753 (0.0%) | 7.09 (4.27-11.78) |
| Respiratory depression | 15 (0.2%) | 5,959 (0.1%) | 3.27 (1.97-5.44) |
| Dependence | 14 (0.2%) | 5,911 (0.1%) | 3.08 (1.82-5.21) |
| Hip surgery | 13 (0.2%) | 3,395 (0.0%) | 4.98 (2.89-8.59) |
| Cold sweat | 13 (0.2%) | 9,697 (0.1%) | 1.74 (1.01-3.00) |
| Panic reaction | 13 (0.2%) | 3,945 (0.0%) | 4.29 (2.49-7.39) |
| Alcoholism | 12 (0.2%) | 2,685 (0.0%) | 5.81 (3.3-10.25) |
| Affect lability | 12 (0.2%) | 4,365 (0.0%) | 3.57 (2.03-6.30) |
| Psychotic symptom | 12 (0.2%) | 1,349 (0.0%) | 11.57 (6.55-20.43) |
| Knee operation | 12 (0.2%) | 5,957 (0.1%) | 2.62 (1.49-4.62) |
| Obsessive-compulsive disorder | 12 (0.2%) | 4,001 (0.0%) | 3.90 (2.21-6.88) |
| Catatonia | 12 (0.2%) | 2,683 (0.0%) | 5.82 (3.30-10.26) |
| Hunger | 11 (0.1%) | 6,681 (0.1%) | 2.14 (1.18-3.87) |
| Dystonia | 11 (0.1%) | 6,003 (0.1%) | 2.38 (1.32-4.31) |
| Poisoning | 11 (0.1%) | 4,825 (0.0%) | 2.96 (1.64-5.36) |
| Dysphoria | 11 (0.1%) | 2,385 (0.0%) | 6.00 (3.32-10.85) |
| Conversion disorder | 10 (0.1%) | 750 (0.0%) | 17.34 (9.29-32.37) |
| Cardiac operation | 10 (0.1%) | 4,923 (0.0%) | 2.64 (1.42-4.91) |
| Hyperventilation | 10 (0.1%) | 2,906 (0.0%) | 4.47 (2.40-8.33) |
| Alcohol poisoning | 10 (0.1%) | 1,200 (0.0%) | 10.84 (5.81-20.20) |
| Depression suicidal | 10 (0.1%) | 1,682 (0.0%) | 7.73 (4.15-14.4) |
| Psychiatric symptom | 9 (0.1%) | 3,965 (0.0%) | 2.95 (1.53-5.68) |
| Screaming | 9 (0.1%) | 3,241 (0.0%) | 3.61 (1.88-6.95) |
| Blood pressure diastolic increased | 9 (0.1%) | 3,090 (0.0%) | 3.79 (1.97-7.29) |
| Depersonalisation/derealisation disorder | 9 (0.1%) | 1,013 (0.0%) | 11.55 (5.99-22.28) |
| Hallucinations, mixed | 9 (0.1%) | 2,044 (0.0%) | 5.72 (2.97-11.02) |
| Hypopnoea | 9 (0.1%) | 2,254 (0.0%) | 5.19 (2.70-9.99) |
| Frustration tolerance decreased | 9 (0.1%) | 5,853 (0.1%) | 2.00 (1.04-3.84) |
| Drug screen positive | 9 (0.1%) | 3,054 (0.0%) | 3.83 (1.99-7.37) |
| Hypomania | 9 (0.1%) | 1,547 (0.0%) | 7.56 (3.93-14.57) |
| Drug monitoring procedure not performed | 9 (0.1%) | 1,320 (0.0%) | 8.87 (4.60-17.08) |
| Hangover | 9 (0.1%) | 2,060 (0.0%) | 5.68 (2.95-10.94) |
| Flashback | 9 (0.1%) | 237 (0.0%) | 49.38 (25.37-96.12) |
| Self-destructive behaviour | 8 (0.1%) | 274 (0.0%) | 37.96 (18.79-76.7) |
| Alcohol abuse | 8 (0.1%) | 1,622 (0.0%) | 6.41 (3.20-12.85) |
| Cystitis interstitial | 7 (0.1%) | 930 (0.0%) | 9.78 (4.65-20.59) |
| Motion sickness | 7 (0.1%) | 1,151 (0.0%) | 7.91 (3.76-16.63) |
| Tearfulness | 7 (0.1%) | 2,121 (0.0%) | 4.29 (2.04-9.01) |
| Shoulder operation | 7 (0.1%) | 2,653 (0.0%) | 3.43 (1.63-7.20) |
| Drug diversion | 7 (0.1%) | 1,761 (0.0%) | 5.17 (2.46-10.86) |
| Attention deficit hyperactivity disorder | 7 (0.1%) | 2,295 (0.0%) | 3.96 (1.89-8.33) |
| Disturbance in social behaviour | 7 (0.1%) | 1,047 (0.0%) | 8.69 (4.13-18.28) |
| Sedation complication | 7 (0.1%) | 1,488 (0.0%) | 6.11 (2.91-12.85) |
| Near death experience | 7 (0.1%) | 2,365 (0.0%) | 3.85 (1.83-8.08) |
| Therapeutic product effect increased | 7 (0.1%) | 816 (0.0%) | 11.15 (5.3-23.47) |
| Bladder pain | 7 (0.1%) | 1,895 (0.0%) | 4.80 (2.29-10.09) |
| Drug monitoring procedure incorrectly performed | 7 (0.1%) | 492 (0.0%) | 18.5 (8.77-39.01) |
| Autism spectrum disorder | 6 (0.1%) | 2,604 (0.0%) | 2.99 (1.34-6.67) |
| Bipolar I disorder | 6 (0.1%) | 947 (0.0%) | 8.24 (3.69-18.38) |
| Agoraphobia | 6 (0.1%) | 712 (0.0%) | 10.95 (4.9-24.47) |
| Nonspecific reaction | 6 (0.1%) | 2,906 (0.0%) | 2.68 (1.20-5.98) |
| Metabolic surgery | 6 (0.1%) | 869 (0.0%) | 8.97 (4.02-20.04) |
| Intrusive thoughts | 6 (0.1%) | 637 (0.0%) | 12.24 (5.48-27.36) |
| Alcohol use | 6 (0.1%) | 1,147 (0.0%) | 6.80 (3.05-15.17) |
| Chemotherapy | 6 (0.1%) | 2,122 (0.0%) | 3.67 (1.65-8.19) |
| Homicidal ideation | 6 (0.1%) | 1,471 (0.0%) | 5.30 (2.38-11.82) |
| Flat affect | 6 (0.1%) | 973 (0.0%) | 8.01 (3.59-17.89) |
| Electric shock sensation | 6 (0.1%) | 1,623 (0.0%) | 4.80 (2.15-10.71) |
| Suicidal behaviour | 6 (0.1%) | 2,044 (0.0%) | 3.81 (1.71-8.50) |
| Intracranial aneurysm | 6 (0.1%) | 2,255 (0.0%) | 3.46 (1.55-7.71) |
| Fear of death | 6 (0.1%) | 1,088 (0.0%) | 7.17 (3.21-15.99) |
| Logorrhoea | 6 (0.1%) | 1,775 (0.0%) | 4.39 (1.97-9.80) |
| Tachyphrenia | 5 (0.1%) | 1,977 (0.0%) | 3.29 (1.37-7.91) |
| Nasal operation | 5 (0.1%) | 446 (0.0%) | 14.57 (6.03-35.19) |
| Substance use disorder | 5 (0.1%) | 1,062 (0.0%) | 6.12 (2.54-14.73) |
| Nasal polyps | 5 (0.1%) | 2,157 (0.0%) | 3.01 (1.25-7.25) |
| Feeling of relaxation | 5 (0.1%) | 338 (0.0%) | 19.23 (7.95-46.5) |
| Feeling guilty | 5 (0.1%) | 616 (0.0%) | 10.55 (4.37-25.44) |
| Aneurysm | 5 (0.1%) | 2,684 (0.0%) | 2.42 (1.01-5.82) |
| Seizure like phenomena | 5 (0.1%) | 1,141 (0.0%) | 5.69 (2.37-13.71) |
| Spinal fusion surgery | 5 (0.1%) | 2,463 (0.0%) | 2.64 (1.10-6.35) |
| Lyme disease | 5 (0.1%) | 2,050 (0.0%) | 3.17 (1.32-7.62) |
| Language disorder | 5 (0.1%) | 1,686 (0.0%) | 3.85 (1.60-9.27) |
| Somatic symptom disorder | 5 (0.1%) | 722 (0.0%) | 9.00 (3.73-21.70) |

Abbreviations: CI, confidence interval; ROR, reporting odds ratio

**Table S3. Sensitivity analysis: disproportionality estimates for 72 AEs with potential safety signals compared to venlafaxine**

| **PT** | **Esketamine  (n = 7790 cases)** | **Venlafaxine**  **(n = 61,441 cases)** | **ROR (95% CI)** |
| --- | --- | --- | --- |
| Dissociation | 1217 (15.6%) | 61 (0.1%) | 186.3 (143.88-241.24) |
| Dissociative disorder | 59 (0.8%) | 11 (0.0%) | 42.62 (22.38-81.16) |
| Sedation | 829 (10.6%) | 270 (0.4%) | 26.98 (23.47-31.02) |
| Drug delivery system malfunction | 15 (0.2%) | 5 (0.0%) | 23.71 (8.61-65.24) |
| Autoscopy | 16 (0.2%) | 8 (0.0%) | 15.8 (6.76-36.94) |
| Incorrect dose administered by device | 48 (0.6%) | 27 (0.0%) | 14.1 (8.8-22.61) |
| Drug monitoring procedure incorrectly performed | 7 (0.1%) | 4 (0.0%) | 13.81 (4.04-47.2) |
| Feeling of relaxation | 5 (0.1%) | 3 (0.0%) | 13.15 (3.14-55.05) |
| Morbid thoughts | 21 (0.3%) | 13 (0.0%) | 12.77 (6.39-25.52) |
| Psychogenic seizure | 17 (0.2%) | 11 (0.0%) | 12.21 (5.72-26.08) |
| Device defective | 16 (0.2%) | 11 (0.0%) | 11.49 (5.33-24.78) |
| Nasal operation | 5 (0.1%) | 4 (0.0%) | 9.86 (2.65-36.74) |
| Chemotherapy | 6 (0.1%) | 5 (0.0%) | 9.47 (2.89-31.04) |
| Therapeutic product effect increased | 7 (0.1%) | 6 (0.0%) | 9.21 (3.09-27.41) |
| Nasal discomfort | 27 (0.3%) | 30 (0.0%) | 7.12 (4.23-11.98) |
| Self-destructive behaviour | 8 (0.1%) | 9 (0.0%) | 7.02 (2.71-18.19) |
| Hospitalisation | 292 (3.7%) | 344 (0.6%) | 6.92 (5.91-8.1) |
| Device malfunction | 65 (0.8%) | 82 (0.1%) | 6.3 (4.54-8.73) |
| Drug monitoring procedure not performed | 9 (0.1%) | 12 (0.0%) | 5.92 (2.49-14.06) |
| Illusion | 16 (0.2%) | 22 (0.0%) | 5.75 (3.02-10.95) |
| Flashback | 9 (0.1%) | 13 (0.0%) | 5.47 (2.34-12.79) |
| Euphoric mood | 74 (0.9%) | 110 (0.2%) | 5.35 (3.98-7.19) |
| Product complaint | 18 (0.2%) | 31 (0.1%) | 4.59 (2.57-8.2) |
| Underdose | 91 (1.2%) | 159 (0.3%) | 4.56 (3.52-5.9) |
| Hip surgery | 13 (0.2%) | 23 (0.0%) | 4.46 (2.26-8.82) |
| Nasal polyps | 5 (0.1%) | 9 (0.0%) | 4.38 (1.47-13.08) |
| Conversion disorder | 10 (0.1%) | 19 (0.0%) | 4.16 (1.93-8.94) |
| Taste disorder | 38 (0.5%) | 75 (0.1%) | 4.01 (2.71-5.93) |
| Near death experience | 7 (0.1%) | 14 (0.0%) | 3.95 (1.59-9.78) |
| Suicidal ideation | 753 (9.7%) | 1675 (2.7%) | 3.82 (3.49-4.18) |
| Motion sickness | 7 (0.1%) | 15 (0.0%) | 3.68 (1.5-9.04) |
| Device issue | 96 (1.2%) | 209 (0.3%) | 3.66 (2.87-4.66) |
| Rehabilitation therapy | 15 (0.2%) | 34 (0.1%) | 3.48 (1.9-6.4) |
| Drug diversion | 7 (0.1%) | 16 (0.0%) | 3.45 (1.42-8.4) |
| Device delivery system issue | 6 (0.1%) | 14 (0.0%) | 3.38 (1.3-8.8) |
| Fear of death | 6 (0.1%) | 14 (0.0%) | 3.38 (1.3-8.8) |
| Cystitis interstitial | 7 (0.1%) | 17 (0.0%) | 3.25 (1.35-7.84) |
| Disturbance in social behaviour | 7 (0.1%) | 17 (0.0%) | 3.25 (1.35-7.84) |
| Adverse event | 81 (1.0%) | 202 (0.3%) | 3.19 (2.46-4.13) |
| Cardiac operation | 10 (0.1%) | 25 (0.0%) | 3.16 (1.52-6.58) |
| Seizure like phenomena | 5 (0.1%) | 13 (0.0%) | 3.03 (1.08-8.52) |
| Feeling drunk | 34 (0.4%) | 91 (0.1%) | 2.96 (1.99-4.39) |
| Major depression | 84 (1.1%) | 234 (0.4%) | 2.85 (2.22-3.66) |
| Hypoaesthesia oral | 24 (0.3%) | 67 (0.1%) | 2.83 (1.77-4.52) |
| Hallucination, visual | 98 (1.3%) | 284 (0.5%) | 2.74 (2.18-3.46) |
| Derealisation | 24 (0.3%) | 70 (0.1%) | 2.71 (1.7-4.31) |
| Product packaging quantity issue | 6 (0.1%) | 18 (0.0%) | 2.63 (1.04-6.63) |
| Panic attack | 163 (2.1%) | 503 (0.8%) | 2.59 (2.17-3.09) |
| Hypertension | 379 (4.9%) | 1211 (2.0%) | 2.54 (2.26-2.86) |
| Hypopnoea | 9 (0.1%) | 28 (0.0%) | 2.54 (1.2-5.38) |
| Spinal operation | 26 (0.3%) | 81 (0.1%) | 2.54 (1.63-3.95) |
| Negative thoughts | 17 (0.2%) | 53 (0.1%) | 2.53 (1.47-4.38) |
| Bladder pain | 7 (0.1%) | 22 (0.0%) | 2.51 (1.07-5.88) |
| Substance abuse | 19 (0.2%) | 61 (0.1%) | 2.46 (1.47-4.12) |
| Blood pressure increased | 345 (4.4%) | 1159 (1.9%) | 2.41 (2.13-2.72) |
| Surgery | 82 (1.1%) | 273 (0.4%) | 2.38 (1.86-3.05) |
| Self-injurious ideation | 22 (0.3%) | 73 (0.1%) | 2.38 (1.48-3.84) |
| Hypertensive crisis | 18 (0.2%) | 62 (0.1%) | 2.29 (1.36-3.88) |
| Neoplasm malignant | 33 (0.4%) | 115 (0.2%) | 2.27 (1.54-3.34) |
| Therapeutic product effect decreased | 48 (0.6%) | 172 (0.3%) | 2.21 (1.6-3.04) |
| Product dose omission issue | 258 (3.3%) | 941 (1.5%) | 2.2 (1.91-2.53) |
| Sensory disturbance | 33 (0.4%) | 119 (0.2%) | 2.19 (1.49-3.23) |
| Paralysis | 19 (0.2%) | 70 (0.1%) | 2.14 (1.29-3.56) |
| Psychotic symptom | 12 (0.2%) | 47 (0.1%) | 2.02 (1.07-3.8) |
| Hyperacusis | 15 (0.2%) | 60 (0.1%) | 1.97 (1.12-3.48) |
| Hallucination | 157 (2.0%) | 658 (1.1%) | 1.9 (1.59-2.27) |
| Product administration error | 22 (0.3%) | 92 (0.1%) | 1.89 (1.19-3.01) |
| Dysgeusia | 61 (0.8%) | 264 (0.4%) | 1.83 (1.38-2.42) |
| Therapeutic response decreased | 43 (0.6%) | 189 (0.3%) | 1.8 (1.29-2.51) |
| Treatment noncompliance | 57 (0.7%) | 255 (0.4%) | 1.77 (1.33-2.36) |
| Unresponsive to stimuli | 34 (0.4%) | 160 (0.3%) | 1.68 (1.16-2.43) |
| Suicide attempt | 248 (3.2%) | 1227 (2.0%) | 1.61 (1.4-1.85) |
| Completed suicide | 168 (2.2%) | 943 (1.5%) | 1.41 (1.2-1.67) |
| Vomiting | 395 (5.1%) | 2445 (4.0%) | 1.29 (1.16-1.44) |
| Depression | 448 (5.8%) | 2912 (4.7%) | 1.23 (1.11-1.36) |
